# Influence of SiO_2_/TiO_2_ Nanocomposite on the Optoelectronic Properties of PFO/MEH-PPV-Based OLED Devices

**DOI:** 10.3390/polym10070800

**Published:** 2018-07-20

**Authors:** Bandar Ali Al-Asbahi

**Affiliations:** 1 Department of Physics & Astronomy, College of Science, King Saud University, Riyadh 11451, Saudi Arabia; alasbahibandar@gmail.com or balasbahi@ksu.edu.sa; Tel.: +966-114-676-620; 2 Research Chair in Laser Diagnosis of Cancers, College of Science, King Saud University, Riyadh 11451, Saudi Arabia

**Keywords:** organic light-emitting diodes (OLEDs), PFO/MEH-PPV hybrids, SiO_2_/TiO_2_ nanocomposite, optoelectronic properties

## Abstract

The influence of SiO_2_/TiO_2_ nanocomposites on the performance of organic light-emitting diodes (OLEDs) based on poly(9,9′-di-n-octylfluorenyl-2,7-diyl) (PFO) and various amounts of poly(2-methoxy-5-(2-ethyl-hexyloxy)-1,4-phenylene-vinylene) (MEH-PPV) was investigated. Prior to the fabrication of the OLEDs on indium-tin oxide (ITO) substrates, the hybrids of PFO/MEH-PPV, in the presence and absence of the SiO_2_/TiO_2_ nanocomposites, were prepared via the solution blending technique. Improvement of the performances of the devices in the presence of the SiO_2_/TiO_2_ nanocomposites was detected. The existence of the SiO_2_/TiO_2_ nanocomposites led to better charge carrier injection and, thus, a significant reduction in the turn-on voltage of the devices. The enhancement of MEH-PPV electroluminescence peaks in the hybrids in the presence of SiO_2_/TiO_2_ nanocomposites is not only a result of the Förster resonance energy transfer, but also of hole-electron recombination, which is of greater significance. Moreover, the existence of the SiO_2_/TiO_2_ nanocomposites led to a shift of the CIE chromaticity coordinates of the devices.

## 1. Introduction

There are many advantages that make conjugated polymers attractive as emissive materials in organic light-emitting diode (OLED) devices. Examples of these advantages are low operating voltage, low cost of fabrication, ease of processing and manufacturing, flexibility, capability to build devices with large-area and good solubility in common organic solvents and photothermal stability [1,2,3,4]. OLEDs with various colors can be achieved by several techniques, such as: (i) building bilayers in a tandem diode structure [5,6]; (ii) using a single polymer with multiple functional groups [7,8]; (iii) blending of conjugated polymers [9,10]; (iv) mixing polymers with nanostructured materials [11,12], quantum dots [13,14] and small phosphorescent [15,16] or fluorescent [8,17] molecules. 

For display applications, polyfluorene (PF) and polyphenylene vinylene (PPV) derivatives have emerged as an attractive class of conjugated polymers because of their good processability and high charge carrier mobility coupled with efficient electroluminescence [18,19]. OLEDs based on hybrids of poly(9,9-di-n-octylfluorenyl-2,7-diyl) (PFO), which acts as a donor material and has a relatively large band gap, and poly(2-methoxy-5-(2-ethyl-hexyloxy)-1,4-phenylene-vinylene) (MEH-PPV), acting as an acceptor material, have attracted research attention [20,21]. By combining two conjugated polymers with contrasting electrical properties, OLEDs based on hybrids of conjugated polymers, as emissive layers, offer numerous advantageous features compared to single component layers, with luminance efficiency being improved by balancing electron and hole injection [22,23]. In spite of these features, hybrid components require further precautions because a material that emits at a lower energy may also absorb in the range of the spectral emission of another material that emits at a higher energy [9,24], resulting in nonradiative energy transfer processes (Förster resonance energy transfer mechanism) [25,26]. The band intensity of the higher energy emission may be reduced or even eliminated when the nonradiative energy transfer processes is very efficient. Nevertheless, both an increase in the luminous performance and color tuning can be achieved concurrently by the careful choice of the concentration of the lower energy material [9] or by incorporation of suitable nanostructure materials in the hybrids [27,28]. Therefore, it is necessary to understand the emission dynamics of the hybrid materials to interpret the performance of the device.

In a recently-published report, it was demonstrated that the emission intensities of pure SiO_2_ and TiO_2_ nanoparticles can be enhanced by mixing them to form SiO_2_/TiO_2_ nanocomposites [29]. This enhancement in the emission intensities can be attributed to the presence of oxygen vacancies and the trapped electrons at the interface of SiO_2_/TiO_2_ nanocomposite thin films [29]. Therefore, it can be hypothesized that the incorporation of SiO_2_/TiO_2_ nanocomposites into the hybrids of PFO/MEH-PPV will lead to distinctive enhancement in OLED device performance.

In the current study, the enhancement of OLED performance based on a hybrid of donor (PFO) and various acceptor (MEH-PPV) amounts in the presence and absence of SiO_2_/TiO_2_ nanocomposites will be characterized in terms of electroluminescence spectra (EL), current-voltage measurements, turn-on voltage and color measurements.

## 2. Materials and Methods

Both poly(2-methoxy-5-(2-ethyl-hexyloxy)-1,4-phenylene-vinylene) (MEH-PPV, MW = 40,000 g/mol) and poly(9,9′-di-n-octylfluorenyl-2.7-diyl) (PFO, MW = 58,200 g/mol) were purchased from Sigma Aldrich, Saint Louis, MO, USA, and used as received without further purification. The SiO_2_/(20%TiO_2_) was prepared as described in a recent report [29]. Prior to fabrication of the thin films, all materials were dissolved in toluene, which was purchased from Fluka (Buchs, Switzerland).

The hybrids of PFO/MEH-PPV with various weight ratios of MEH-PPV (0.1, 0.5, 1.0, 5.0 and 10 wt.%), in the presence and absence of a fixed ratio of 10% SiO_2_/(20% TiO_2_) nanocomposites, were prepared by the solution blending technique. Before fabrication of the OLEDs, the indium-tin oxide (ITO) substrates (1.2 cm × 2 cm) were treated as reported in recent work [30]. Then, the prepared materials were employed as emissive layers by deposition onto ITO substrates using spin coating (2000 rpm for 20 s), followed by annealing at 120 °C in a vacuum oven to remove the solvent. To deposit an aluminum cathode, the ITO substrates with the emissive layers were moved to an electron beam chamber, where the deposition rate was 2 Å/min at a chamber pressure of 2.5 ×10^−6^ Pa. For all the OLED devices, the thickness of the fabricated Al cathode was 150 nm, and the active area of each device was 0.076 cm^2^.

A Keithley 238 measurement system (Cleveland, OH, USA) was used for I-V measurements, and an HR2000 Ocean Optics Spectrometer (Metric Drive, FL, USA) was used for EL and color measurements.

## 3. Results and Discussion

### 3.1. Current-Voltage Measurements

**Figure 1:**
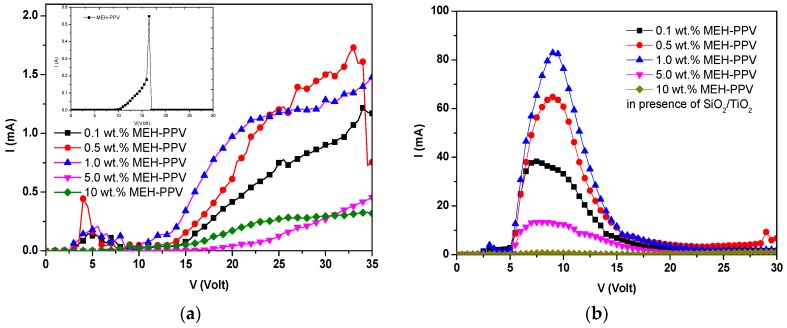
Current-voltage (I-V) measurements of the OLEDs based on hybrids of PFO/MEH-PPV. (**a**) In the absence of SiO_2_/TiO_2_ nanocomposites; (**b**) in the presence of SiO_2_/TiO_2_ nanocomposites. The inset shows the I-V curve of the OLEDs based on pristine MEH-PPV.

Figure 1 shows the current-voltage (I-V) characteristics of pristine MEH-PPV and PFO/MEH-PPV hybrid-based OLED devices in both the absence and presence of SiO_2_/TiO_2_ nanocomposites. It can be observed that the current increased in the presence of the SiO_2_/TiO_2_ nanocomposites, while the turn-on voltage decreased, which demonstrated the improvement of the performance of the OLED device. The higher voltage (>10 V) led to enhancement of the light emissive layer resistivity, and then, the currents rapidly decreased. Nevertheless, the incorporation of SiO_2_/TiO_2_ nanocomposites resulted in an increase in the current of more than 40-times compared to that measured in their absence. 

The higher current can be attributed to a reduction in the resistance and activation energy of the emissive layer [20,31]. The lower turn-on voltage of injection current in the presence of SiO_2_/TiO_2_ nanocomposites compared to their absence can be attributed to a better charge carrier injection [28,32].

However, the gradual reduction in current upon incrementing the MEH-PPV content (more than 1.0 wt.%) indicated the high resistivity of the devices. Once the content of MEH-PPV exceeded 1 wt.% within the hybrids, more holes and electrons were trapped, and consequently, lower current (and higher resistivity) was observed. This current reduction can be ascribed to MEH-PPV trapping both holes and electrons in PFO and instantaneously holding back their transport. Many reports demonstrate that lower current (and higher resistivity) may cause higher efficiency in exciton confinement and hole-electron recombination, which are crucial for better OLED device performance [33,34,35].

### 3.2. Electroluminescence Spectra

**Figure 2:**
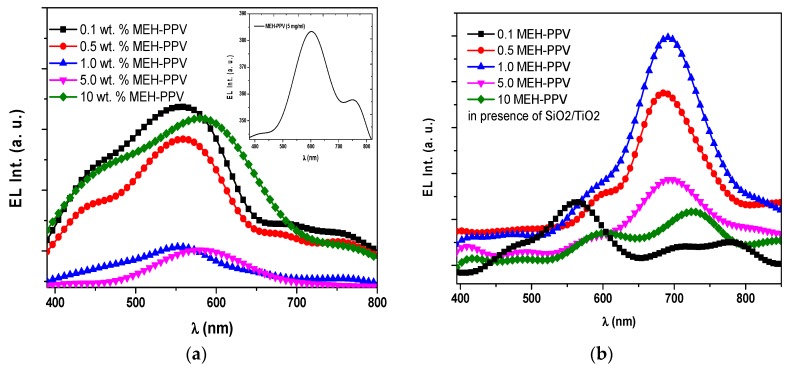
Electroluminescence (EL) spectra of the OLEDs based on PFO/MEH-PPV hybrids at applied voltages corresponding to maximum luminance. (**a**) In the absence of SiO_2_/TiO_2_ nanocomposites; (**b**) in the presence of SiO_2_/TiO_2_ nanocomposites. The inset shows the EL spectra of the OLEDs based on pristine MEH-PPV.

Figure 2 presents the EL spectra of OLED devices based on pristine MEH-PPV and PFO/(0.1, 0.5, 1.0, 5.0, 10 wt.%) MEH-PPV hybrids in the absence and presence of SiO_2_/TiO_2_ nanocomposites. The EL spectra of all OLED devices were dominated by the MEH-PPV emission with a lesser contribution from PFO emission at 440 nm. The profile does not match the behavior observed in the fluorescence spectra (not shown here), where the contribution from the emission of PFO was much more pronounced. In the case of SiO_2_/TiO_2_ nanocomposites being present, a significant observation in EL spectra can be detected when the content of MEH-PPV exceeded 0.1 wt.%. The peak assigned to the MEH-PPV (740 nm) was enhanced and red-shifted with increasing MEH-PPV content, whereas the peak intensity at 550 nm was decreased. By comparing the EL spectra with the fluorescence spectra in all the OLED devices, the relative EL intensity peak of the PFO diminished significantly upon incrementing the MEH-PPV content. Such significant differences in fluorescence and EL spectra for hybrids of polyfluorene derivatives have been reported [9,36,37]. Moreover, the significant difference between the fluorescence and EL spectra was strong evidence that Förster resonance energy transfer (FRET) was not the only mechanism occurring in the OLED devices during the EL measurements [5]. Whilst fluorescence spectra are created via various types of energy transfer processes or direct excitation, the EL spectra strongly depend on additional factors such as charge transport, charge injection from the electrodes, recombination processes and exciton generation. 

A cascade mechanism can be proposed for charge injection in the hybrids that prefers exciton formation in a lower energy gap polymer. The strong relative decrease of the PFO spectrum contribution to the EL spectra for all the OLED devices suggested that after the injection of holes and into the OLED device, either the recombination favorably happened in the MEH-PPV monomers or the excitons were primarily generated in this polymer phase. 

Since the HOMO and LUMO of MEH-PPV lay within the range of those of PFO [20,38], it is possible that exciton formation in MEH-PPV occurred through the cascade mechanism. Moreover, due to the position of the MEH-PPV, there was a strong probability that it may have been acting as a trap for the charge carriers and then enhanced charge recombination in this phase. This probability was also evidenced significantly by the reduction in turn-on voltage, as discussed in the section above.

It can be concluded that in the EL spectra, the dominance of the peaks assigned to MEH-PPV in the hybrids was not only the result of FRET, but also arose from hole-electron recombination, which is of greater significance.

### 3.3. Color Measurements

**Figure 3:**
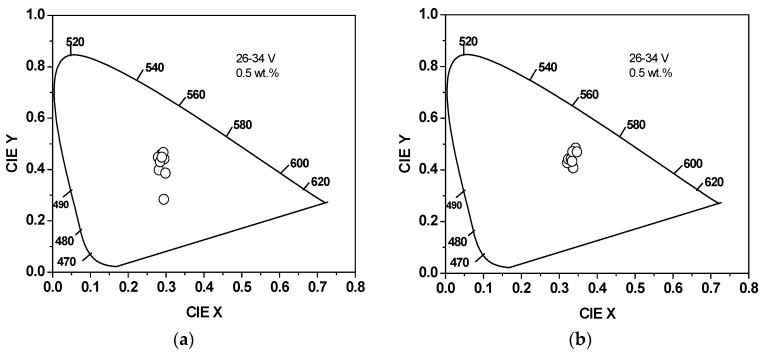
CIE coordinates of the OLEDs based on PFO/MEH-PPV hybrids when the applied voltage was increased from 26–34 V. (**a**) In the absence of SiO_2_/TiO_2_ nanocomposites, (**b**) in the presence of SiO_2_/TiO_2_ nanocomposites.

Figure 3 shows the CIE coordinates of the PFO/0.5 wt.% MEH-PPV-based OLED device in the absence and presence of SiO_2_/TiO_2_ nanocomposites when the applied voltage was varied from 26–34 V. A blue-shift in the emitted color of the OLEDs was detected with increasing voltage due to phase separation. Many researchers have shown that mixing of two polymers with different emission and charge-transport properties leads to a shift in the color of emission of the OLEDs with varying operating voltage [39,40]. Submicrometer-sized domains with a range of compositions can be caused by phase separation. Subsequently, some of the excited states can be created in the polymer with the higher band gap and then lost to the lower band gap polymer by exciton transfer. The existence of the blue-shifted colors at higher voltages was due to the fact that the electron-hole injection in the higher energy gap required a higher field [40]. 

Table 1 summarizes the CIE chromaticity coordinates of the OLEDs based on PFO/MEH-PPV hybrids, with and without SiO_2_/TiO_2_ nanocomposites, at the applied voltage that caused the highest luminance. The slight shift in CIE coordinate values upon the increment of the MEH-PPV content confirmed that the produced color was stable and consistent with the EL spectra.

Moreover, the existence of SiO_2_/TiO_2_ nanocomposites played a crucial role in shifting CIE chromaticity coordinates with respect to those in their absence. This finding is in agreement with the EL spectra (Figure 2), which were red-shifted in the presence of SiO_2_/TiO_2_ nanocomposites. This shifting can be attributed to efficient Förster resonance energy transfer in the presence of SiO_2_/TiO_2_ nanocomposites and also to the extension of hole-electron recombination zones [11].

## 4. Conclusions

The solution blending technique was successfully employed to prepare PFO/MEH-PPV hybrids, in the presence and absence of SiO_2_/TiO_2_ nanocomposites, which were used as the emissive layer in OLED devices. The incorporation of SiO_2_/TiO_2_ nanocomposites into the hybrids played a crucial role in enhancing the optoelectronic properties of the devices. The improvement of the performance of the OLED device is demonstrated by the significant reduction in turn-on voltage and the increase in the current of the devices in the presence of SiO_2_/TiO_2_ nanocomposites compared to those values in their absence. This improvement in the device performance started to reduce when the MEH-PPV content exceeded 1.0 wt.%, even in the presence of the SiO_2_/TiO_2_ nanocomposites, because MEH-PPV trapped both holes and electrons in the PFO. The shift of EL spectra and CIE coordinates upon addition of SiO_2_/TiO_2_ nanocomposites illustrated efficient Förster resonance energy transfer and extension of the recombination zone for holes and electrons in the OLED devices.

## Figures and Tables

**Table 1:** The CIE chromaticity coordinates delivered by the OLEDs at the applied voltage, which caused highest luminance.

Acceptor Content in the Blend (wt. %)	In the Presence of SiO_2_/TiO_2_ Nanocomposite			In the Absence of SiO_2_/TiO_2_ Nanocomposite		
	CIE Coordinates		V (Volt)	CIE Coordinates		V (Volt)
	X	Y		X	Y	
0.1	0.313	0.395	31.5	0.294	0.284	36
0.5	0.337	0.447	30.5	0.298	0.305	34
1	0.333	0.413	29.5	0.285	0.37	39.5
5	0.318	0.279	34	0.374	0.346	34.5
10	0.307	0.226	39.5	0.319	0.243	38

## References

[1] Assaka A.M., Rodrigues P.C., De Oliveira A.R., Ding L., Hu B., Karasz F.E., Akcelrud L. (2004). Novel fluorine containing polyfluorenes with efficient blue electroluminescence. Polymer.

[2] Liu S.P., Chan H.S., Ng S.C. (2004). Poly[2,7-(9,9-dihexylfluorene)-alt-pyridine] with donor-acceptor architectures: A new series of blue-light-emitting alternating copolymers. J. Polym. Sci. A Polym. Chem..

[3] Jokinen K., Bykov A., Sliz R., Remes K., Fabritius T., Myllylä R. (2015). Luminescence and spectrum variations caused by thermal annealing in undoped and doped polyfluorene OLEDs. Solid-State Electron..

[4] Cuerva C., Campo J.A., Cano M., Arredondo B., Romero B., Otón E., Otón J.M. (2015). Bis(pyridylpyrazolate)platinum(ii): A mechanochromic complex useful as a dopant for colour-tunable polymer OLEDs. New J. Chem..

[5] De Azevedo D., Freitas J.N., Domingues R.A., Faleiros M.M., Atvars T.D.Z. (2017). Correlation between the PL and EL emissions of polyfluorene-based diodes using bilayers or polymer blends. Synth. Met..

[6] Zhao D., Liu H., Miao Y., Wang H., Zhao B., Hao Y., Zhu F., Xu B. (2016). A red tandem organic light-emitting diode based on organic photovoltaic-type charge generation layer. Org. Electron..

[7] Nicolai H.T., Hof A., Blom P.W. (2012). Device Physics of White Polymer Light-Emitting Diodes. Adv. Funct. Mater..

[8] Reineke S., Thomschke M., Lüssem B., Leo K. (2013). White organic light-emitting diodes: Status and perspective. Rev. Mod. Phys..

[9] Quites F.J.N., Faria G.R.C., Germino J.C., Atvars T.D.Z. (2014). Tuning emission colors from blue to green in polymeric light-emitting diodes fabricated using polyfluorene blends. J. Polym. Chem..

[10] Baek S.J., Chang H.J. (2012). Fabrication and characterization of white polymer light emitting diodes using PFO: MDMO-PPV. J. Nanosci. Nanotechnol..

[11] Al-Asbahi B.A., Jumali M.H.H., Yap C.C., Salleh M.M. (2013). Influence of TiO_2_ nanoparticles on enhancement of optoelectronic properties of PFO-based light emitting diode. J. Nanomater..

[12] Chen D., Liang J., Pei Q. (2016). Flexible and stretchable electrodes for next generation polymer electronics: A review. Sci. China Chem..

[13] Borriello C., Prontera C., Mansour S.A., Aprano S., Maglione M., Bruno A., Luccio T.D., Minarini C. (2015). Optoelectronic properties of OLEDs based on CdSe/ZnS quantum dots and F8BT. Phys. Status Solidi C.

[14] Kang B.-H., You T.-Y., Yeom S.-H., Kim K.-J., Kim S.-H., Lee S.-W., Yuan H., Kwon D.-H., Kang S.-W. (2012). Highly efficient white light-emitting diodes based on quantum dots and polymer interface. IEEE Photon. Technol. Lett..

[15] Ikawa S., Yagi S., Maeda T., Nakazumi H. (2012). White polymer light-emitting diodes co-doped with phosphorescent iridium complexes bearing the same cyclometalated ligand. Phys. Status Solidi C.

[16] Ouyang X., Li X.-L., Bai Y., Mi D., Ge Z., Su S.-J. (2015). Highly-efficient hybrid white organic light-emitting diodes based on a high radiative exciton ratio deep-blue emitter with improved concentration of phosphorescent dopant. RSC Adv..

[17] Derue L., Olivier S., Tondelier D., Maindron T., Geffroy B., Ishow E. (2016). All-solution-processed organic light-emitting diodes based on photostable photo-cross-linkable fluorescent small molecules. ACS Appl. Mater. Interfaces.

[18] Amorim C., Cavallari M., Santos G., Fonseca F.J., Andrade A., Mergulhão S. (2012). Determination of carrier mobility in MEH-PPV thin-films by stationary and transient current techniques. J. Non-Cryst. Solids..

[19] Scherf U., List E.J. (2002). Semiconducting polyfluorenes—Towards reliable structure–property relationships. Adv. Mater..

[20] Bajpai M., Srivastava R., Kamalasanan M., Tiwari R., Chand S. (2010). Charge transport and microstructure in PFO: MEH-PPV polymer blend thin films. Synth. Met..

[21] Chen H.C., Wang C.T., Liu C.L., Liu Y.C., Chen W.C. (2009). Full color light-emitting electrospun nanofibers prepared from PFO/MEH-PPV/PMMA ternary blends. J. Polym. Sci. B Polym. Phys..

[22] Buckley A., Rahn M., Hill J., Cabanillas-Gonzalez J., Fox A., Bradley D. (2001). Energy transfer dynamics in polyfluorene-based polymer blends. Chem. Phys. Lett..

[23] Grice A., Bradley D., Bernius M., Inbasekaran M., Wu W., Woo E. (1998). High brightness and efficiency blue light-emitting polymer diodes. Appl. Phys. Lett..

[24] Sahoo H. (2011). Förster resonance energy transfer–A spectroscopic nanoruler: Principle and applications. J. Photochem. Photobiol. C.

[25] Főrster T. (1959). 10th Spiers Memorial Lecture. Transfer mechanisms of electronic excitation. Discuss. Faraday Soc..

[26] Gupta V., Bharti V., Kumar M., Chand S., Heeger A.J. (2015). Polymer–Polymer Förster Resonance Energy Transfer Significantly Boosts the Power Conversion Efficiency of Bulk-Heterojunction Solar Cells. Adv. Mater..

[27] Al-Asbahi B.A., Jumali M.H.H., Yap C.C., Salleh M.M., AlSalhi M.S. (2013). Inhibition of dark quenching by TiO2 nanoparticles content in novel PFO/fluorol 7GA hybrid: A new role to improve OLED performance. Chem. Phys. Lett..

[28] Al-Asbahi B.A., Haji Jumali M.H., AlSalhi M.S. (2016). Enhanced optoelectronic properties of PFO/Fluorol 7GA hybrid light emitting diodes via additions of TiO_2_ nanoparticles. Polymers.

[29] Al-Asbahi B.A. (2017). Influence of anatase titania nanoparticles content on optical and structural properties of amorphous silica. Mater. Res. Bull..

[30] Al-Asbahi B.A. (2017). Energy transfer mechanism and optoelectronic properties of (PFO/TiO_2_)/Fluorol 7GA nanocomposite thin films. Opt. Mater..

[31] Chang C.-C., Hsieh M.-T., Chen J.-F., Hwang S.-W., Chen C.H. (2006). Highly power efficient organic light-emitting diodes with ap-doping layer. Appl. Phys. Lett..

[32] Jumali M.H.H., Al-Asbahi B.A., Yap C.C., Salleh M.M., Alsalhi M.S. (2012). Optoelectronic property enhancement of conjugated polymer in poly (9, 9′-di-n-octylfluorenyl-2.7-diyl)/titania nanocomposites. Thin Solid Films.

[33] Zhang Q., Chambers D.K., Selmic S. (2006). Polymer Light-Emitting Diodes Based on Poly [9, 9-di-(2′-ethylhexyl) fluorenyl-2.7-diyl]. J. Nanoelectron. Optoe..

[34] Hsieh S.-N., Kuo T.-Y., Hsu P.-C., Wen T.-C., Guo T.-F. (2007). Study of polymer blends on polymer light-emitting diodes. Mater. Chem. Phys..

[35] Al-Asbahi B.A., Jumali M.H.H., Yap C.C., Flaifel M.H., Salleh M.M. (2013). Photophysical properties and energy transfer mechanism of PFO/Fluorol 7GA hybrid thin films. J. Lumin..

[36] De Deus J.F., Faria G.C., Iamazaki E.T., Faria R.M., Atvars T.D., Akcelrud L. (2011). Polyfluorene based blends for white light emission. Org. Electron..

[37] Voigt M., Chappell J., Rowson T., Cadby A., Geoghegan M., Jones R.A., Lidzey D.G. (2005). The interplay between the optical and electronic properties of light-emitting-diode applicable conjugated polymer blends and their phase-separated morphology. Org. Electron..

[38] Li Y., Cao Y., Gao J., Wang D., Yu G., Heeger A.J. (1999). Electrochemical properties of luminescent polymers and polymer light-emitting electrochemical cells. Synth. Met..

[39] Berggren M., Inganäs O., Gustafsson G., Andersson M.R., Hjertberg T., Wennerström O. (1995). Controlling colour by voltage in polymer light emitting diodes. Synth. Met..

[40] Berggren M., Inganäs O., Gustafsson G., Rasmusson J., Andersson M.R., Hjertberg T., Wennerström O. (1994). Light-emitting diodes with variable colours from polymer blends. Nature.

